# CRABP2 affects chemotherapy resistance of ovarian cancer by regulating the expression of HIF1α

**DOI:** 10.1038/s41419-023-06398-4

**Published:** 2024-01-09

**Authors:** Xin Fu, Qian Zhang, Zhaosong Wang, Yue Xu, Qiuping Dong

**Affiliations:** 1 Tianjin Medical University Cancer Institute and Hospital, National Clinical Research Center for Cancer, Tianjin, 300060 China; 2 Key Laboratory of Cancer Prevention and Therapy, Tianjin, 300060 China; 3 Tianjin’s Clinical Research Center for Cancer, Tianjin, 300060 China; 4 Department of Gynecologic Oncology, Tianjin Medical University Cancer Institute and Hospital, Tianjin, 300060 China; 5 Medical Affairs Office, Tianjin Cancer Hospital Airport Hospital, Tianjin, 300060 China; 6 Laboratory Animal Center, Tianjin Medical University Cancer Institute and Hospital, Tianjin, 300060 China; 7 Laboratory of Tumor Cell Biology, Tianjin Medical University Cancer Institute and Hospital, Tianjin, 300060 China

**Keywords:** Ovarian cancer, Drug development, Drug regulation

## Abstract

Ovarian cancer is the most lethal malignancy among gynecologic cancers, and primary and secondary chemotherapy resistance is one of the important reasons for poor prognosis of ovarian cancer patients. However, the specifics of resistance to chemotherapy in ovarian cancer remain unclear. Herein, we find that the expression level of cellular retinoic acid binding protein 2 (CRABP2) is up-regulated in drug-resistant ovarian cancer tissues and cell lines, and the expression levels of CRABP2 in epithelial ovarian cancer tissues are closely related to tumor clinical stage and patients’ prognosis, suggesting that CRABP2 plays an important role in the progression of ovarian cancer and the corresponding ability of tumor to chemotherapy. With the in-depth study, we demonstrates that CRABP2 is related to the high metabolic activity in drug-resistant cells, and all-trans retinoic acid exacerbates this activity. Further molecular mechanism exploration experiments show that CRABP2 not only up-regulates the expression level of HIF1α, but also increases the localization of HIF1α in the nucleus. In drug-resistant ovarian cancer cells, knocking down HIF1α can block the resistance of CRABP2 to chemotherapy drugs in ovarian cancer cells. Taken together, our findings suggest for the first time that CRABP2 affects chemotherapy resistance of ovarian cancer by regulating the expression of HIF1α. This study provides a possible molecular mechanism for drug resistance and a possible molecular target for clinical treatment of ovarian cancer.

## Introduction

Ovarian cancer is the most lethal malignancy among the three major gynecologic cancers, with the third-highest incidence rate in gynecologic tumors [1]. However, it has the highest mortality rate, posing a serious threat to the life and health of female patients [2]. Due to its location in the abdominal cavity, early symptoms of ovarian cancer are insidious, and approximately 75% of patients are diagnosed at an advanced stage with widespread metastasis, where simple surgery cannot eliminate the tumor [3]. Therefore, surgery combined with platinum-based chemotherapy is a commonly used clinical treatment for ovarian cancer. Although most ovarian cancer patients are relatively sensitive to platinum-based chemotherapy in the early stages, more than 60% of patients experience recurrence [4]. Furthermore, many patients develop secondary drug resistance after chemotherapy, which directly leads to the inability of chemotherapy drugs to effectively inhibit ovarian cancer cells, resulting in disease progression and seriously affecting the survival and quality of life of ovarian cancer patients [5–9]. Therefore, ovarian cancer drug resistance has become a difficult problem in clinical treatment, and the study of its mechanisms is particularly important, as these studies will provide new ideas for reversing ovarian cancer drug resistance.

Cellular retinoic acid binding protein 2 (CRABP2) belongs to the retinoic acid binding protein family and the lipid calcium-binding protein/cytosolic fatty acid binding protein family, and is mainly involved in retinoic acid (RA) signal transduction [10]. RA is a critical regulatory factor in gene expression during embryonic development, playing an important role in cell growth and differentiation [11]. CRABP2 is a nuclear shuttle protein that can transport RA from cytoplasm to nucleus, in which RA interacts with nuclear receptors (RAR/RXR), and regulate gene expression [12]. In addition, CRABP2 can interact with HuR, enhancing the binding ability of HuR to target RNA and increasing RNA stability [13]. Currently, abnormal expression of CRABP2 has been found in various tumors [14–17], either upregulated or downregulated, and its role in tumors is not yet clear. The expression of CRABP2 in breast cancer tissues is significantly up-regulated, and it may become a new diagnostic marker for breast cancer [18]. The expression level of CRABP2 in uterine fibroids is significantly higher than that in normal uterine smooth muscle, and estrogen can promote the expression of CRABP2, suggesting that CRABP2 plays an important role in the occurrence of uterine fibroids [19]. The expression level of CRABP2 in liver cancer tissue increases with tumor progression, and inhibiting the expression of CRABP2 can suppress the proliferation, migration, and invasion of tumor cells in vitro [16]. I In addition, CRABP2 promotes lung cancer cell metastasis through HuR and integrin β1/FAK/ERK signaling, and plasma CRABP2 is being used as a novel biomarker in patients with non-small cell lung cancer [20, 21]. However, in head and neck squamous cell carcinoma, CRABP2 is significantly downregulated due to high methylation and its loss of expression is associated with reduced disease-free survival in patients [22]. Therefore, the regulation of CRABP2 expression may vary in different tumors and exhibit significant tissue specificity.

Currently, there is limited research on CRABP2 in ovarian cancer. One study found that the exosome-related CRABP2 is upregulated and promotes cell proliferation in ovarian cancer, and CRABP2 performs better than the currently used biomarker, CA125, in the diagnosis of ovarian cancer [23]. Another research showed that CRABP2 promotes the proliferation and invasion of ovarian cancer cells by upregulating the expression of EZH2 [24]. In this study, we found that CRABP2 was related to the sensitivity of ovarian cancer cells to chemotherapy drugs, and the expression level of CRABP2 in drug-resistant ovarian cancer cells was significantly upregulated. Meanwhile, inhibition of CRABP2 expression can significantly reverse tumor drug resistance. Mechanistically, we found that abnormal expression of CRABP2 can cause abnormal activity of the RA signaling pathway, leading to upregulation of HIF1α expression and increased overall metabolic activity in cells, ultimately resulting in ovarian cancer cell drug resistance. Our study provides a new idea for the study of drug resistance of ovarian cancer, and provides an experimental basis for clinical treatment of ovarian cancer.

## Materials and methods

### Patients and tissue samples

The public databases GSE1926 and GSE15709 were used to analyze genes that might influence drug sensitivity of ovarian cancer. The public database KmPlot was used to study the relationships between CRABP2 mRNA expression levels and prognosis of patients with ovarian cancer, and the cutoff with the highest hazard (HR) rate was selected for the final analysis. Gene Expression Profiling Interactive Analysis 2 (GEPIA2) data and the Human Protein Atlas (HPA) were used to analyze the expression levels of CRABP2 in ovarian tissues, ovarian cancer tissues and different types of ovarian cancer tissues. In addition to the public databases about ovarian cancer, 128 epithelial ovarian cancer (EOC) tissues were obtained from the Department of Pathology, Tianjin Medical University Cancer Institute and Hospital. All tissues were examined by specialists using the World Health Organization criteria. Determination of cancer stage and grade was according to the International Federation of Gynecology and obstetrics. The use of these specimens and patient’s information was approved by the Ethics Committee of Tianjin Medical University Cancer Institute and Hospital.

### Drug resistance related clinical and pathological parameters

In 128 EOC patients, there were 103 receiving chemotherapy (paclitaxel/cisplatin or Taxo/cisplatin). 103 patients were divided into two groups, sensitive group and partial sensitive group (64 cases), and chemotherapy resistant group (39 cases) according to the guideline of National Comprehensive Cancer Network (recurrence beyond 12 months or didn’t recurrence after the chemotherapy was defined as sensitive group, recurrence between 6 to 12 months after the chemotherapy was partial sensitive group, recurrence during the chemotherapy period or within 6 months after the chemotherapy was drug resistance group).

### Immunohistochemistry staining

In brief, tissue sections were firstly deparaffinized and graded with xylene and alcohol solutions, and then exposed antigen by high pressure heat repair in 10 mM citrate buffer (pH 6.0) and blocked endogenous peroxidase by 3% hydrogen peroxide solution. Then sections were incubated with primary antibody CRABP2 (1:100, Cat#ab181255, abcam) or HIF1α (1:100, Cat#ab51608, abcam) for overnight at 4°C, and incubated with secondary antibody for 1 h at 37°C following with DAB staining for 5 min to visualize immunolabeling. The expression levels of CRABP2 or HIF1α were obtained by multiplying the percentage of positive staining score with the staining intensity score. Percentage points is defined as: 1 (0% ~ 25%), 2 (26% ~ 50%), 3 (51% ~ 75%), 4 (76% ~ 100%). Intensity scores were defined as: 0 (no staining), 1 (low staining), 2 (moderate staining), and 3 (high staining). According to the final score, all tissues were divided into high expression group (score ≥6) and low expression group (score < 6).

### Ascites tumor cell isolation

Ascitic fluid was collected from the peritoneal cavity of the ovarian cancer patient through paracentesis. About 100 ml of ascitic fluid were collected under ultrasound guidance from each ovarian cancer patient with confirmed ascites, then the collected ascitic fluid was immediately transferred to the laboratory for pre-filtration with a 70 μm cell filter. The filtered ascitic fluid was centrifuged at a speed of 1000 r/min for 10 mins to separate the cellular components. Next the precipitated cells were gently mixed with 1 mL of pre-cooled red cell lysis buffer and incubated at room temperature for 10 minutes. Then 10 mL of calcium-free and magnesium-free pre-cooled PBS was added and centrifuged at a speed of 1000 r/min for 10 mins. The sedimented cells were washed twice with calcium-free and magnesium-free PBS and once with RPMI 1640 medium supplemented with 10% of fetal bovine serum (Hyclone, USA), 100 U/ml of penicillin, and 100 μg/ml of streptomycin (Invitrogen, USA). After adjusting the appropriate cell concentration, the cells were placed in 96-well plates for subsequent cisplatin treatment.

### Cell culture and treatment

Human EOC cell lines (SKOV3 and A2780) were purchased from ATCC (American Type Culture Collection), and cultured in RPMI 1640 medium supplemented with 10% of fetal bovine serum, 100 U/ml of penicillin, and 100 μg/ml of streptomycin. SKOV3/DDP and A2780/DDP cells were purchased from Wuhan Procell Life Science & Technology Co., Ltd. (Wuhan, China). 1 μg/mL DDP and 0.6 μg/mL DDP was added to the drug-resistant cell culture medium of SKOV3/DDP and A2780/DDP cells, respectively, to maintain drug resistance. The culture medium was renewed with DDP-free medium three days before the experiment. Cells were grown at 37°C in a humidified incubator with 5% CO_2_. All cell lines were tested for Mycoplasma contamination and authenticated by short tandem repeat profiling. Cisplatin (DDP, P4394) and All-trans retinoic acid (ARTA, R2625) were obtained from Sigma-Aldrich.

### Plasmid construction and cell infection

In this study, small hairpin RNAs (shRNA) were constructed into pLKO.1 plasmids to down-regulate CRABP2 or HIF1α. The shRNA sequences were as follows: shCRABP2: GCACCACAGAGAUUAACUUTT; shHIF1α: GTGATGAAAGAATTACCGAAT. Regarding the construction of the overexpressed CRABP2 plasmid, full-length CRABP2 was amplified by PCR using the following primers: upper: 5ʹ-GAGATCTAGAGCCACCATGCCCAACTT

CTCTGGCAACTGGAAAATC-3ʹ, lower: 5ʹ-GAGAGAATTCCTCTCGGACGTAGACCCTG

GTGCACACAAC-3ʹ, and cloned into the pCDH-CMV-MCS-Puro lentiviral vector. All the plasmids were further confirmed by DNA sequencing. HEK293T cells were used for lentivirus production by co-transfecting with the target plasmid and two packaging plasmids (psPAX2 and pMD2.G) through polyethyleneimine system. After 48 h of transfection, the virus-containing supernatant was collected and used to infect cancer cells. Real-time PCR and western blot were used to verify the infect efficiency.

### Quantitative Real-Time PCR

Total RNA was extracted by Trizol, and reverse transcribed into cDNA by HiScript II Q RT SuperMix for qPCR (Vazyme, China). Then PCR was performed using AceQ qPCR SYBR Green Master Mix (Vazyme, China) according to the manufacturer’s protocol. The sequences of primers used for real-time PCR are listed as follows: CRABP2 (forward): 5-GATGCCTCTTGCAGGGTCTT-3, CRABP2 (reverse): 5-GATGCCTCTTGCAGGGTCTT-3; HIF1α (forward): 5-TGCTCATCAGTTGCCACTTC-3, HIF1α (reverse): 5-TGGGCCATTTCTGTGTGTAA-3; β-actin (forward): 5-GAAATCGTGCGTGACATTAA-3, β-actin (reverse): 5-AAGGAAGGCTGGAAGAGTG-3. The gene expression was calculated by the 2-ΔΔCT method.

### Western blot analysis

1 × SDS ice-cold lysis buffer was used to prepare cell lysates, and BCA (bicinchoninic acid) method was used to determine the protein concentration. Proteins were resolved on a denaturing 10% SDS-PAGE gel, subsequently transferred to polyvinylidene fluoride membranes (PVDF) via semidry transfer. The PVDF was then blocked in 5% dried milk in Tris-buffered saline and Tween 20, incubated with primary antibodies CRABP2 (1:1 000), HIF1α (1:1 000) and β-actin (1:5 000, Cat#ab181602, abcam) for overnight at 4 °C, and then with secondary antibodies for 1 h at 37 °C. Signals were detected using the enhanced chemiluminescence (ECL) luminol reagent (Millipore, MA, USA). Image J was used to calculate the grayscale and β-actin protein was used as a loading control.

### MTT assay for cell viability and proliferation

MTT assay was performed with Roche Cell proliferation Kit to detect cell viability and proliferation. In 100 µL cell medium, cells were seeded on a 96-well plate at a density of 10^4^ cells/well and incubate for 48 h. After the incubation period, 10 μL MTT labeling reagent was added to each well, and incubated for 4 h in a humidified incubator. Then 100 μL Solubilization buffer was added into each well, and absorbance was read at 570 nm.

### Flow cytometry

Annexin V-FITC/PI Apoptosis Detection Kit (Vazyme, China) was used to detect the cell death by flow cytometry. The cells were first inoculated into a 6-well plate and treated with chemotherapy drugs for 48 h after the cells were attached to the wall. The cells were then collected in 200 μL buffer and stained with 5 μL Annexin V-FITC and 5 μL PI solution. The stained cells were analyzed using flow cytometry (BD, franklin lakes, New Jersey, USA).

### ATP level detection

CellTiter-Glo Luminescent Cell Viability Assay kit (G7570, Promega) was used to determine ATP levels of cells according to manufacturer’s instructions. Simply, 5 × 10^4^ cells of every group were plated to the two 24-well plates, and the cells of the one 24-well plate were lysed with ATP assay lysis buffer 24 h later. Then incubated at room temperature for 10 minutes, and the supernatant was collected to measure total ATP levels based the luminous signals. Meanwhile, cells of the other 24-well plate were used to count the number of cells at the time of ATP harvest, and then the ATP luminescent value and cell number of each group were homogenized.

### Quantification of Glucose and lactate

L-Lactate Assay Kit (K627-100) and Glucose Assay Kit (K606-100) were purchased from BioVision Technology Company. 1 × 10^5^ cells of every group were plated at the same volume to 12-well plate, then culture media were collected 24 h later and detected concentrations of lactate and glucose according to manufacturer’s instructions. The concentrations in these samples were determined with reference to the standard curves of lactate and glucose, meanwhile, the number of cells counted at the time of culture-medium harvest was used to normalize the data.

### Seahorse assay

OCR and ECAR were detected by an XF24 Extracellular Flux Analyzer (Agilent). The assays were performed according to manufacturer’s instructions. Specifically, cells were seeded in 24-well plates at a density of 4 × 10^4^ cells per well in a growth medium overnight. Assay was performed with a following protocol: three measurements were taken for each stage, and media was mixed for 3 min and the measurement lasted 3 min for each measurement. For OCR analysis, cells were incubated with a base medium containing 10 mM glucose, 2 mM L-glutamine and 1 mM pyruvate. After every three measurements at 6 min intervals, 1 μM oligomycin, 5 μM FCCP, or 1 μM rotenone was added to the wells at the indicated time points. For ECAR analysis, cells were added base medium in the presence of 2 mM L-glutamine and monitored every 6 min following successive administration of 10 mM glucose, 1 μM oligomycin, and 2 mM 2-deoxyglucose.

### Luciferase Activity Analysis

Promega Dual-Luciferase™ Reporter (DLR™) Assay Systems was used according to the manufacturer’s instructions, and HIF1α reporter plasmid and control plasmid were constructed by Gene-Chem (Shanghai Genechem Co.,Ltd.). The cells were seeded into 96-well plates at a density of 2 × 10^3^ cells/well and transfected with corresponding plasmids. After 24 h, cells were incubated for 24 h in presence or absence of ATRA. Then the cells were lysed, and firefly and renilla activities were determined. The luciferase signal was normalized using the renilla values.

### Mice xenograft model

Female Balb/c-nu/nu mice, age 5–6 weeks and weighed 17–18 g, were obtained from the Beijing HFK Bioscience Co., and housed in a pathogen free environment (12 h light and dark cycles) at Laboratory Animal Center of Tianjin Medical University Cancer Institute and Hospital. 5×10^6^ SKOV3/DDP cells of shctrl or shCRABP2 were injected subcutaneously to the right of the dorsal midline in mice to generate murine subcutaneous tumors. When the subcutaneous tumor grew to about 5 mm in diameter (about 10 days later), the mice were randomly divided into two groups, one group was given normal saline, and the other group was given cisplatin (2 mg/kg) by intraperitoneal injection every day. Tumor formation and tumor size of mice were observed regularly. At the end of the experiment, mice were killed, tumor nodules were removed, and their volume and weight were measured. The animal experiment was terminated at 3 weeks, then the subcutaneous tumor was dissected and the tumor volume was measured. Tumor volumes were calculated by the following formula: length × width × width/2. All animal studies were followed an approved protocol by the Tianjin cancer hospital, in accordance with the principles and procedures outlined in the NIH Guide for the Care and Use of Laboratory Animals.

### Statistical methods

SPSS 19.0 was used to evaluate the data. The standard two-tailed *t*-test was used to assess the expression levels of CRABP2 in different group of EOCs. Kaplan-Meier analysis was performed to analyze the association of CRABP2 expression and survival of patients with EOC. The significance level was defined as p < 0.05.

## Results

### CRABP2 is associated with the prognosis of ovarian cancer patients

**Fig. 1:**
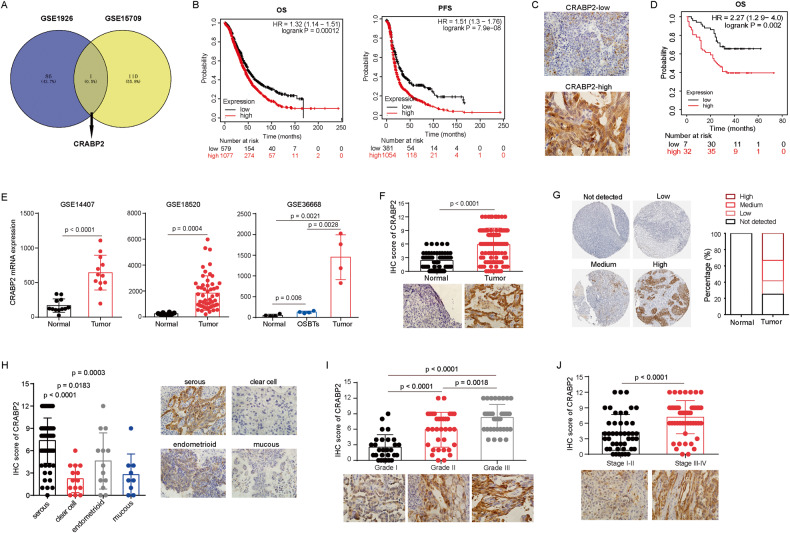
The expression of CRABP2 in ovarian cancer tissues and its relationship with clinicopathological features of patients. **A** The upregulated genes in ovarian cancer drug-resistant cells from the public databases GSE1926 and GSE15709. **B** The relationship between CRABP2 mRNA expression level and the prognosis of ovarian cancer patients in data of KmPlot. **C** The representative pictures of CRABP2 IHC staining. **D** The relationship between CRABP2 protein expression level and the prognosis of ovarian cancer patients. **E** CRABP2 mRNA expression levels in ovarian cancer and normal ovarian tissues of GSE datasets. **F** CRABP2 protein expression levels in ovarian cancer and normal ovarian tissues. G, The CRABP2 protein expression levels in normal ovarian tissues and ovarian cancer tissues in the Human Protein Atlas (HPA). **H** The expression levels of CRABP2 protein in different pathological types of ovarian cancer. **I** The expression levels of CRABP2 protein in different pathological grade of ovarian cancer. **J** The expression levels of CRABP2 protein in different stage of ovarian cancer.

To explore the specific molecular mechanisms underlying chemotherapy resistance in ovarian cancer, we analyzed the upregulated genes in ovarian cancer drug-resistant cells from the public databases GSE1926 and GSE15709, and found that CRABP2 was upregulated in both drug-resistant datasets (Fig. 1A), suggesting that CRABP2 may play an important role in the development of drug resistance. Subsequently, we analyzed the relationship between CRABP2 and the prognosis of ovarian cancer patients using data of KmPlot, and found that CRABP2 was a significant risk factor for OS and PFS (Fig. 1B). We also conducted survival analysis in patients with different clinical stages (Fig. S1A and B) and pathological grades (Fig. S1C and D), and found that regardless of early or late stage, high or low differentiation, CRABP2 was a risk factor for OS and PFS in ovarian cancer patients. Since serous ovarian cancer is the main type of ovarian cancer, we separately analyzed the relationship between CRABP2 and the prognosis of serous ovarian cancer patients, and found that CRABP2 was also a risk factor for OS and PFS in serous ovarian cancer patients (Fig. S1E and F). To further analyze the relationship between CRABP2 and the prognosis of ovarian cancer patients, we used immunohistochemistry to detect the protein expression level of CRABP2 in tumor tissues from 128 patients with serous ovarian cancer (Fig. 1C). The analysis showed that patients with high expression of CRABP2 had a poorer prognosis (Fig. 1D), and multivariate survival analysis showed that CRABP2 was an independent prognostic factor for serous ovarian cancer patients (Table 1). These results indicate that the expression level of CRABP2 in tumor tissues is closely related to the prognosis of ovarian cancer patients.

**Table 1:** Multivariate survival analysis.

Variables	HR	95.0% CI for HR		*P*
		Lower	Upper	
Age, years (<55 vs. ≥55)	1.45	0.87	2.42	0.15
**Stage (I/II vs. III/IV)**	0.13	0.04	0.39	0.00
Grade (well vs. poor)	1.36	0.82	2.24	0.23
Ascites (No vs. Yes)	0.84	0.47	1.50	0.55
**Metastases (Negative vs. Positive)**	2.65	0.99	7.03	0.05
**CRABP2 (Low vs. High)**	2.26	1.27	4.04	0.01

### CRABP2 is highly expressed in ovarian cancer tissues and is associated with the malignancy of the tumor

To better understand the role of CRABP2 in the development and progression of ovarian cancer, we compared its expression levels in normal and ovarian tissues. Gene Expression Profiling Interactive Analysis 2 (GEPIA2) data showed that compared to normal tissues, the expression level of CRABP2 in various cancers is inconsistent, either upregulated or downregulated, with the highest upregulation observed in ovarian cancer (Fig. S2A and B). Multiple GSE datasets also showed that CRABP2 mRNA expression levels are significantly higher in ovarian cancer than in normal ovarian tissue (Fig. 1E). Consistent with RNA expression levels, our immunohistochemistry results demonstrated that CRABP2 protein expression was also significantly higher in ovarian cancer tissues than in corresponding normal ovarian tissues (Fig. 1F). Meanwhile, the Human Protein Atlas (HPA) detection results indicated that CRABP2 protein expression was barely detectable in normal ovarian tissues, but was expressed in ovarian cancer tissues (Fig. 1G). Furthermore, we found that the expression levels of CRABP2 in serous ovarian cancer was higher than in other types of ovarian cancer (Fig. 1H), which was confirmed by the HPA (Fig. S2C). Additionally, the expression level of CRABP2 is positively correlated with the histopathological grade (Fig. 1I) and clinical stage (Fig. 1J) of the tumor. GEPIA2 data also shows that the higher the clinical stage of ovarian cancer, the higher the expression level of CRABP2 mRNA in tumor tissues (Fig. S2D). Moreover, the expression level of CRABP2 mRNA is highest in ovarian cancer metastases (Fig. S2E). These findings demonstrate that the expression level of CRABP2 is closely related to the progression of ovarian cancer.

### The expression level of CRABP2 is related to the sensitivity of ovarian cancer cells to chemotherapy drugs

**Fig. 2:**
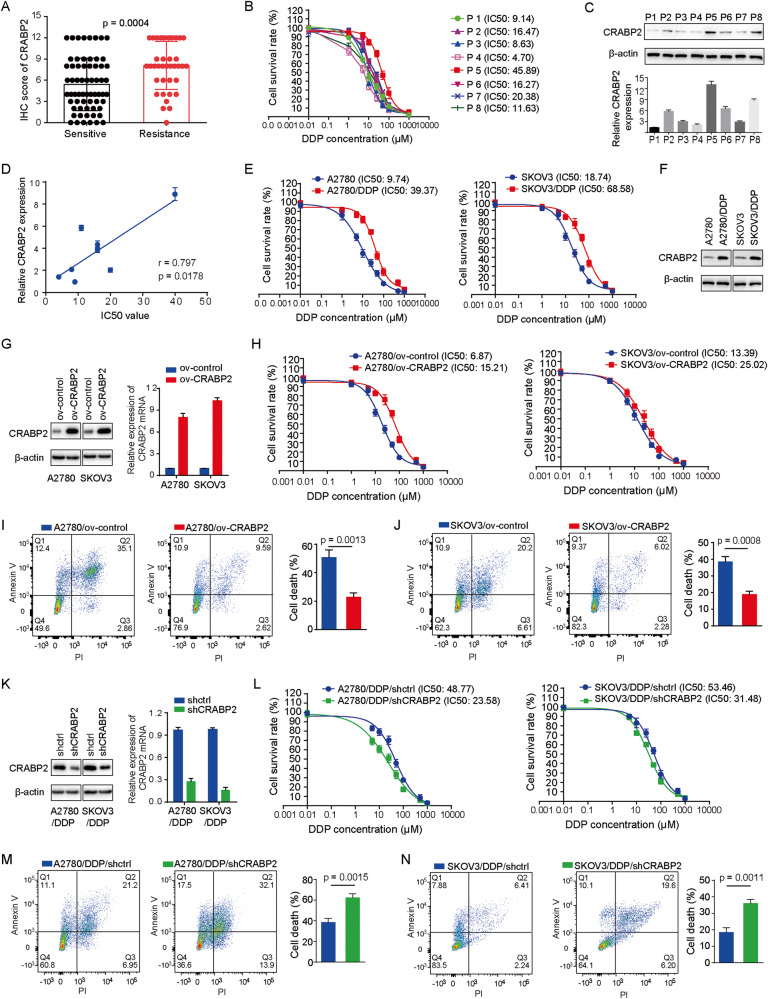
The expression level of CRABP2 is related to the sensitivity of ovarian cancer cells to chemotherapy drugs. **A** The relationship between the expression level of CRABP2 and the sensitivity of patients to chemotherapy. **B** IC50 value of cisplatin in ovarian cancer cells of ascites was determined. **C,** The CRABP2 expression levels of ovarian cancer cells of ascites. **D** The relationship of CRABP2 expression levels and IC50 values of cisplatin in ovarian cancer cells of ascites. **E** IC50 values of cisplatin in ovarian cancer resistant cells and parental cells. **F** The CRABP2 expression levels of ovarian cancer resistant cells and parental cells. **G**, Cell lines overexpressing CRABP2 were constructed in ovarian cancer parental cells. **H** IC50 values of cisplatin in CRABP2 overexpressing and control cells. **I** and **J**, The cell death induced by 10 μM DDP in control cells and overexpressed CRABP2 cells of A2780 (**I**) and SKOV3 (**J**) for 48 h was detected by flow cytometry. **K** CRABP2 knockdown cell lines were constructed in drug-resistant ovarian cancer cells. **L** IC50 values of cisplatin in CRABP2 knockdown and control cells. **M** and **N** The cell death induced by 50 μM DDP in control and CRABP2 knockdown cells of A2780/DDP (**M**) and SKOV3/DDP (**N**) for 48 h.

As mentioned above, CRABP2 was highly expressed in drug-resistant ovarian cancer cells in the database, which made us wonder whether the correlations between CRABP2 higher expression and malignant progression of ovarian cancer and poor prognosis of patients was related to drug resistance. To test this hypothesis, we first analyzed the relationship between the expression level of CRABP2 and the sensitivity of patients to chemotherapy in 128 ovarian cancer patients, and the results showed that the tumor tissues that were not sensitive to chemotherapy had a high level of CRABP2 expression (Fig. 2A). Then we isolated tumor cells from the ascites of eight ovarian cancer patients, and detected their sensitivity to cisplatin (Fig. 2B) and CRABP2 expression levels (Fig. 2C), which showed that CRABP2 expression levels were positively correlated with the IC50 values (Fig. 2D). Subsequently, we found that the expression level of CRABP2 was significantly higher in cisplatin-resistant A2780 and SKOV3 cells than in their parental cells (Fig. 2E and F). Moreover, upregulating the expression of CRABP2 in A2780 and SKOV3 parental cells (Fig. 2G) could suppress their sensitivity to cisplatin (Fig. 2H-J), while inhibiting the expression of CRABP2 (Fig. 2K) could significantly reverse drug resistance in resistant cells (Fig. 2L-N). These results indicate that CRABP2 is closely related to the drug sensitivity of ovarian cancer. KmPlot’s analysis also revealed that among all ovarian cancer patients receiving chemotherapy, those with higher levels of CRABP2 expression in tumor tissue had a poorer prognosis (Fig. S3). Based on the above experimental results, we believe that the expression of CRABP2 does affect the chemotherapy sensitivity of ovarian cancer.

### ATRA affects the sensitivity of ovarian cancer-resistant cells to chemotherapy drugs, but does not affect the corresponding parental cells

**Fig. 3:**
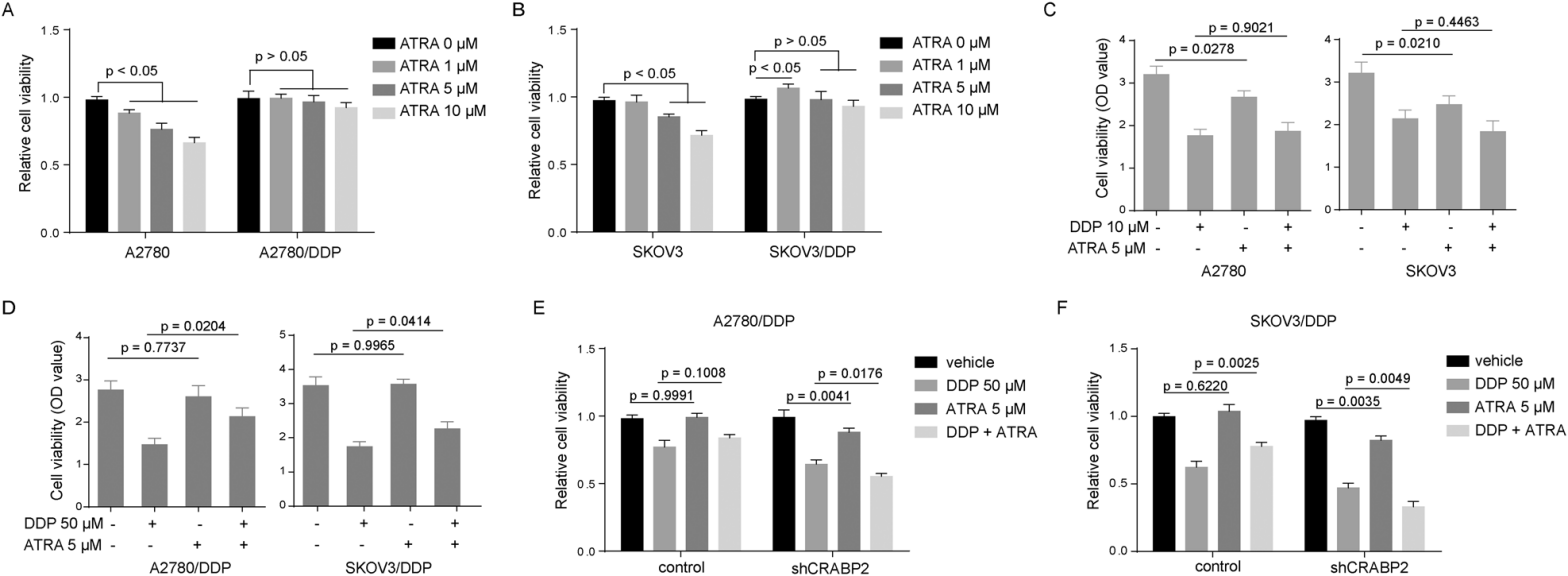
ATRA affects the sensitivity of ovarian cancer-resistant cells to chemotherapy drugs. **A** and **B** Effect of ATRA at different concentrations on the viability of ovarian cancer drug-resistant cells and parental cells. **C** and **D** Effect of ATRA on the sensitivity of ovarian cancer drug-resistant cells (**D**) and parental cells (**C**) to DDP. **E** and **F**, Effects of CRABP2 knockdown on ATRA and DDP sensitivity in A2780/DDP (**E**) and SKOV3/DDP (**F**).

CRABP2 is a specific transporter of retinoic acid (RA), so we wanted to know whether RA can affect chemotherapy sensitivity of ovarian cancer cells, and whether drug sensitivity of ovarian cancer regulated by CRABP2 is related to RA. We treated ovarian cancer-resistant cells and their corresponding parent cells with different concentrations of all-transRA (ATRA), and then measured the viability of these cells. Interestingly, ATRA can inhibit the viability of parental cells, but not resistant cells (Fig. 3A and B). Moreover, ATRA not only cannot increase the sensitivity of ovarian cancer cells to cisplatin, but also inhibits the sensitivity of resistant cells to chemotherapy drugs (Fig. 3C and D). In addition, silencing the expression of CRABP2 in drug-resistant cells increased the sensitivity of these cells to ATRA and increased the sensitization of ATRA to DDP (Fig. 3E, F).

### CRABP2 affects the overall metabolic activity of ovarian cancer cells

**Fig. 4:**
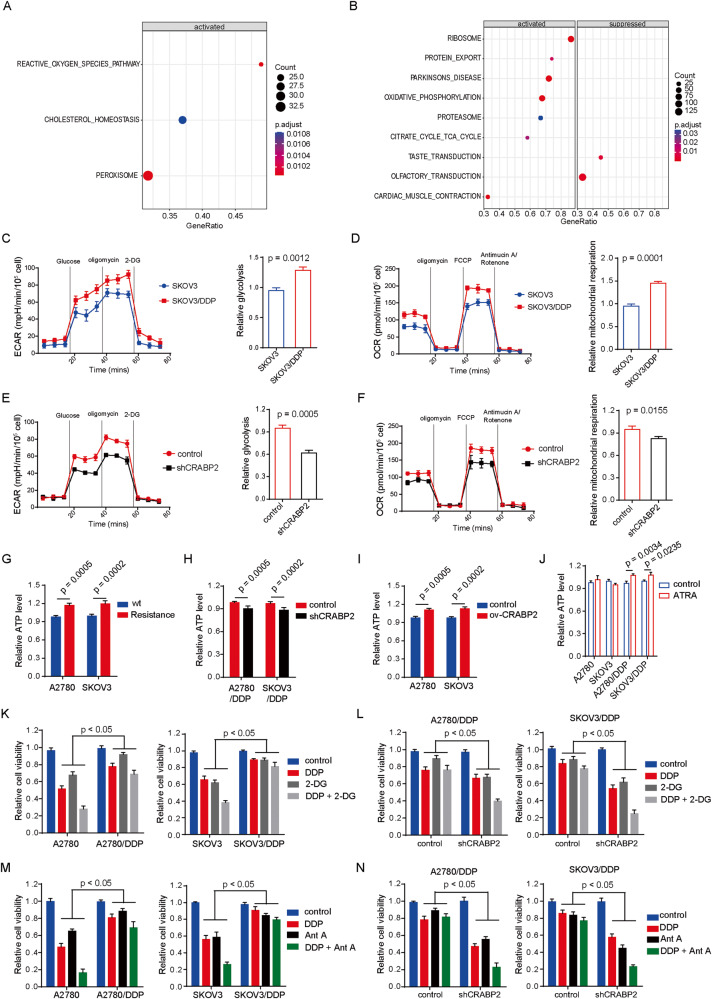
CRABP2 affects the overall metabolic activity of ovarian cancer cells. **A** and **B**, GSEA enrichment analysis (**A**) and KEGG enrichment analysis (**B**) based on the data of ovarian cancer in TCGA. C and D, ECAR (**C**) and OCR (**D**) in SKOV3 and SKOV3/DDP cells detected by a cell energy metabolism analyzer. E and F, ECAR (**E**) and OCR (**F**) in CRABP2 knockdown cells of SKOV3/DDP and control cells. **G**, ATP levels in ovarian cancer drug-resistant cells and parental cells. **H** and **I** Effects of CRABP2 knockdown (**H**) or overexpressing (**I**) on ATP levels in ovarian cancer drug-resistant cells. **J** Effects of ATRA on ATP levels in ovarian cancer drug-resistant cells and parental cells. **K** Effect of 2-DG (10 mM) on the sensitivity of ovarian cancer drug-resistant cells and parental cells to DDP. **L** Effects of CRABP2 knockdown on 2-DG (10 mM) and DDP sensitivity in ovarian cancer drug-resistant cells. **M** Effect of Ant A (2 μM) on the sensitivity of ovarian cancer drug-resistant cells and parental cells to DDP. **N** Effects of CRABP2 knockdown on Ant A (2 μM) and DDP sensitivity in ovarian cancer drug-resistant cells.

To clarify the mechanism by which CRABP2 affects the sensitivity of ovarian cancer cells to chemotherapy drugs, we conducted enrichment analysis on gene data from TCGA database. The GSEA enrichment analysis results showed that high expression of CRABP2 is closely related to the activation of the ROS pathway and cholesterol synthesis pathway (Fig. 4A). The KEGG enrichment analysis results showed that high expression of CRABP2 is related to the activity of oxidative phosphorylation and the tricarboxylic acid cycle (Fig. 4B), suggesting that CRABP2 may be involved in the metabolic regulation of ovarian cancer cells. Subsequently, we used a cell energy metabolism analyzer to detect the lactate production rate and oxygen consumption rate of ovarian cancer cells and their resistant cells. The results showed that the lactate production rate (Fig. 4C) and oxygen consumption rate (Fig. 4D) of resistant cells were higher than those of parental cells, indicating that the overall metabolic capacity and potential of resistant cells were higher than those of parental cells. Knocking down the expression of CRABP2 in resistant cells significantly inhibited their originally high metabolic activity (Fig. 4E and F). ATP is the energy source for all cell activities, and we found that ATP levels were higher in resistant cells than in parental cells (Fig. 4G). Knocking down the expression of CRABP2 in resistant cells significantly inhibited ATP levels (Fig. 4H), while upregulating the expression of CRABP2 in parental cells increased ATP levels (Fig. 4I). ATRA increased ATP levels in ovarian cancer resistant cells but had no effect on ATP levels in parental cells (Fig. 4J). In addition, we found that the glycolysis inhibitor 2-DG and the oxidative phosphorylation inhibitor AntA, which originally had inhibitory effects on parental cell activity, had significantly reduced effects on resistant cell activity (Fig. 4K and M). The combined effect of cisplatin on cell activity was also significantly reduced (Fig. 4 K and M). However, knocking down the expression of CRABP2 in resistant cells significantly enhanced the inhibitory effect of 2-DG and AntA on cell viability (Fig. 4L and N). We speculate that this is because CRABP2 upregulates the activity of oxidative phosphorylation and the tricarboxylic acid cycle, and inhibiting one may be compensated for by the other. Therefore, using 2-DG or AntA alone may not effectively inhibit the activity of resistant cells. These results suggest that CRABP2 may affect the sensitivity of ovarian cancer cells to chemotherapy drugs by increasing the overall metabolic level in these cells.

### CRABP2 affects the metabolism of ovarian cancer cells by regulating the expression of HIF1α

**Fig. 5:**
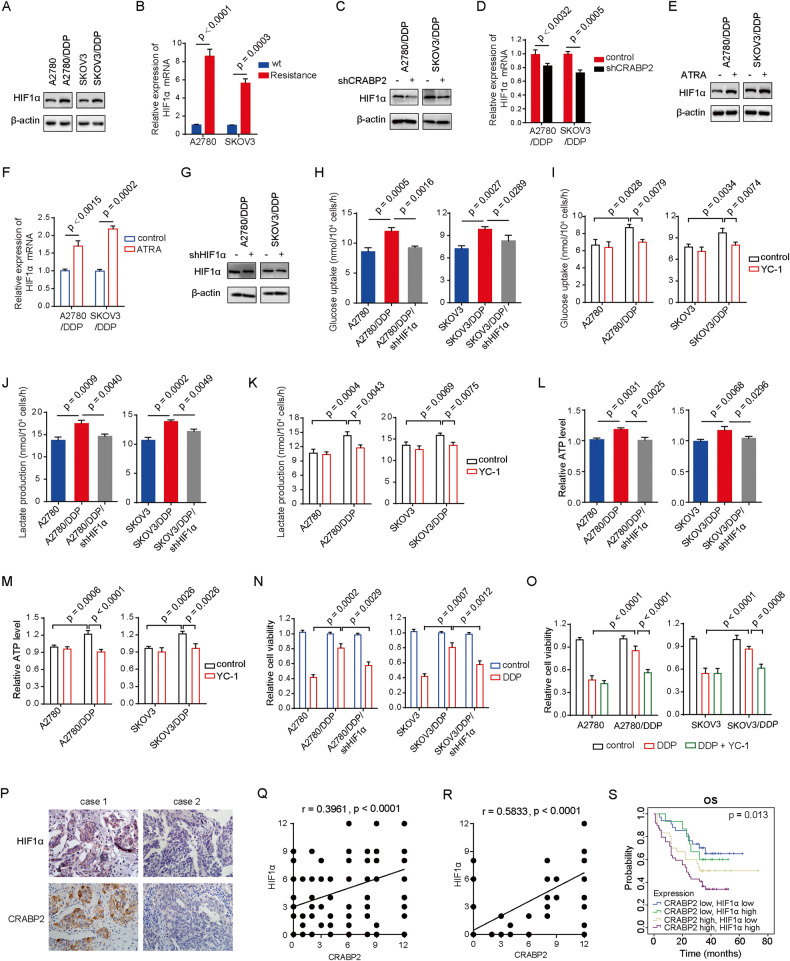
CRABP2 affects the metabolism of ovarian cancer cells by regulating the expression of HIF1α. **A** and **B** HIF1α protein expression levels (**A**) and mRNA levels (**B**) in ovarian cancer drug-resistant cells and parental cells. C and D, Effects of CRABP2 knockdown on HIF1α protein expression levels (**C**) and mRNA levels (**D**) in ovarian cancer drug-resistant cells. **E** and **F**, Effects of ATRA on HIF1α protein expression levels (**E**) and mRNA levels (**F**) in ovarian cancer drug-resistant cells. **G**, HIF1α knockdown cell lines were constructed in ovarian cancer drug-resistant cells. **H** and **I**, Effect of HIF1α knockdown (**H**) or HIF1α inhibitor YC-1 (50 μM) (**I**) on glucose uptake in ovarian cancer drug-resistant cells. J and **K** Effect of HIF1α knockdown (**J**) or HIF1α inhibitor YC-1 (50 μM) (**K**) on lactate production in ovarian cancer drug-resistant cells. **L** and **M**, Effect of HIF1α knockdown (**L**) or HIF1α inhibitor YC-1 (50 μM) (**M**) on ATP levels in ovarian cancer drug-resistant cells. N and O, Effect of HIF1α knockdown (**N**) or HIF1α inhibitor YC-1 (50 μM) (**O**) on the sensitivity of ovarian cancer cells to DDP. P, The representative IHC staining pictures of CRABP2 and HIF1α in same patients. **Q** and **R**, A positive correlation between the protein expression levels of CRABP2 and HIF1α in ovarian cancer tissues (**Q**) and in chemotherapy-insensitive ovarian cancer tissues (**R**). **S** Effect of simultaneous expression of CRABP2 and HIF1α on ovarian cancer patient prognosis.

Currently, it is well established that HIF1α is closely related to the metabolic phenotype of tumor cells, and its expression is involved in regulating various biological activities of tumors, including drug resistance. Based on the previous experimental results, we wanted to know whether CRABP2 could affect the metabolism of ovarian cancer cells by regulating the expression of HIF1α, ultimately affecting the sensitivity of tumor cells to chemotherapy drugs. Our results showed that HIF1α is indeed highly expressed in ovarian cancer-resistant cells (Fig. 5A and B), and knocking down the expression of CRABP2 led to a decrease in both HIF1 protein and RNA levels (Fig. 5C and D). Meanwhile, we found that ATRA increased the protein and RNA expression of HIF1α (Fig. 5E and F). Knocking down HIF1α (Fig. 5G) or using the HIF1α inhibitor YC-1 in resistant cells significantly inhibited glucose uptake and utilization, reduced lactate production, and decreased ATP levels (Fig. 5H-M). Treatment with cisplatin showed that knocking down HIF1α significantly increased cell sensitivity to cisplatin (Fig. 5N). In drug resistant cells overexpressing CRABP2, YC-1 increased cell sensitivity to cisplatin (Fig. 5O). By inhibiting HIF1α with YC-1, the increase in metabolic activity caused by CRABP2 overexpression was significantly inhibited (Fig. S4A-C). Similarly, the increase in metabolic activity caused by ATRA was also significantly restrained by YC-1 (Fig. S4D-F). And YC-1 also inhibited drug resistance in ovarian cancer cells induced by overexpression of CRABP or addition of ATRA (Fig. S4G, H). These results indicate that CRABP2 can affect the expression of HIF1α, and the resistance of CRABP2 depends on the expression of HIF1α. In addition, we found a positive correlation between the protein expression levels of CRABP2 and HIF1α in ovarian cancer tissues (Fig. 5P and Q), and the correlation was stronger in chemotherapy-insensitive ovarian cancer tissues (Fig. 5R). Importantly, ovarian cancer patients with high expression of both CRABP2 and HIF1α in tumor tissues had the worst prognosis (Fig. 5S).

In order to further analyze the specific mechanism by which CRABP2 regulates HIF-1a expression, Actinomycin D was used to detect the stability of HIF1a mRNA. The results showed that neither of the silenced CRABP2 expression nor the addition of ATRA could significantly change the stability of HIF1α mRNA (Fig. S5A, B) Moreover, the analysis results in the database of ENCORI (The Encyclopedia of RNA Interactomes) also showed that the RNA binding protein profile of HIF1α mRNA did not contain CRABP2 (Supplementary excel 1). Subsequently, we analyzed the promoter sequence of HIF1α and found several possible RARE (retinoic acid response element) motifs (Fig. S5C). Then we constructed the HIF1α luciferase reporter plasmid, and found that the luciferase level significantly decreased when CRABP2 was knockdown in drug-resistant cells (Fig. S5D), while ATRA significantly increased the luciferase intensity significantly (Fig. S5E). These results suggest that CRABP2 promotes HIF1α expression by affecting its transcription level.

### CRABP2 affects the subcellular localization of HIF1α

**Fig. 6:**
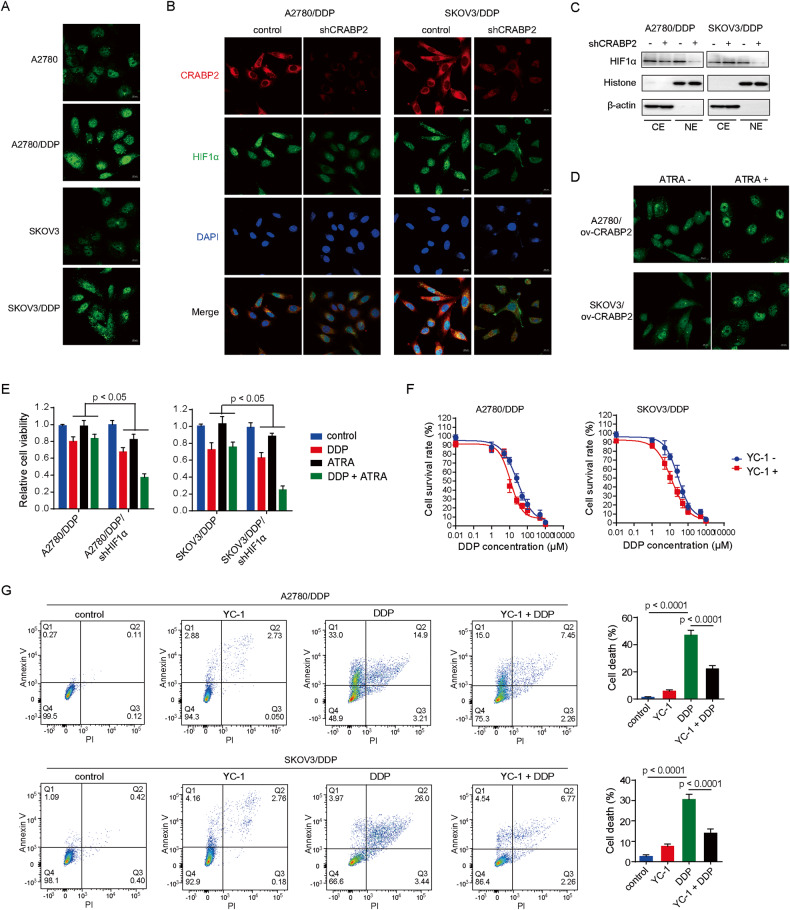
CRABP2 affects the subcellular localization of HIF1α. **A** The expression of CRABP2 in ovarian cancer drug-resistant cells and parental cells detected by immunofluorescence. **B** and **C**, Effects of CRABP2 knockdown on HIF1α expression level and localization in ovarian cancer drug-resistant cells detected by immunofluorescence (**B**) and nuclear-cytoplasmic protein separation (**C**). **D** Effects of ATRA on HIF1α localization in CRABP2 overexpression cells. **E**, Effect of DDP combined with ATRA on the viability of HIF1α-knockdown resistant ovarian cancer cells. **F** Effect of YC-1 on DDP IC50 in drug-resistant ovarian cancer cells. **G** The effect of YC-1 combined with DDP on the death of drug-resistant ovarian cancer cells was detected by flow cytometry.

In addition to finding a positive correlation between the expression levels of CRABP2 and HIF1α, we also found that when CRABP2 was primarily localized in the nucleus, more HIF1α also accumulated in the nucleus. Immunofluorescence results in ovarian cancer cells showed that HIF1α was more prominently localized in the nucleus of resistant cells (Fig. 6A), and knocking down the expression of CRABP2 significantly reduced the level of nuclear HIF1α (Fig. 6B). Results from nuclear-cytoplasmic protein separation experiments also showed that CRABP2 can affect the co-localization of HIF1α in cells (Fig. 6C). At the same time, ATRA can increase the accumulation of HIF1α in the cell nucleus (Fig. 6D). Interestingly, ovarian cancer resistant cells that were originally insensitive to DDP and ATRA showed significantly reduced activity when HIF1α expression was downregulated and treated with DDP in combination with ATRA (Fig. 6E). YC-1 also increased the sensitivity of resistant cells to DDP (Fig. 6F, G). These results suggest that the combined inhibition of HIF1α by ATRA may reverse chemotherapy resistance in ovarian cancer.

### Effect of CRABP2 on drug sensitivity of ovarian cancer cells in mice

**Fig. 7:**
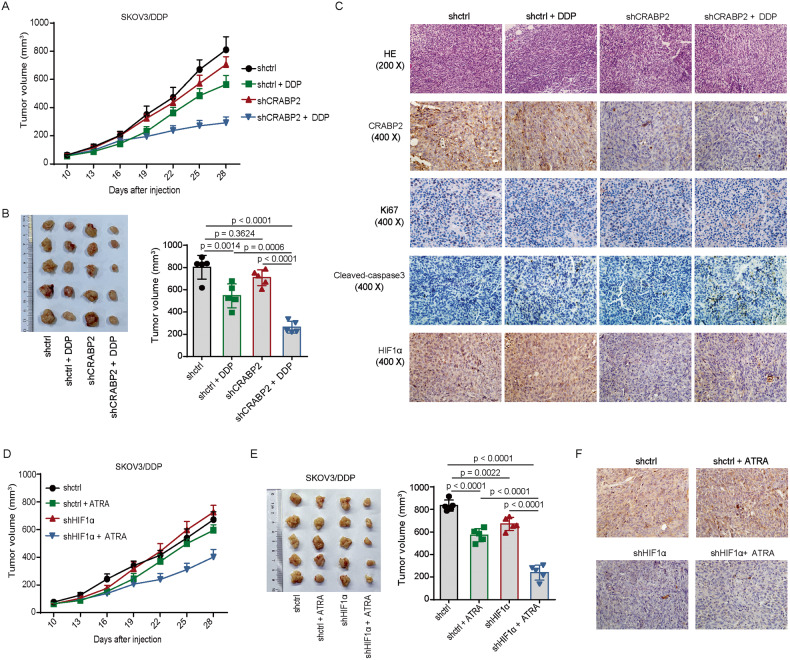
Effect of CRABP2 on drug sensitivity of ovarian cancer cells in mice. **A** Effect of CRABP2 silencing combined with DDP on the growth of tumors in the subcutaneous xenograft tumor model; *n* = 5 for each group. **B** The tumor volumes of mice in each group. **C** Representative images of HE staining and IHC staining for CRABP2, Ki67, Cleaved-caspase 3 and HIF1α in tumor of each group. **D**, Effect of HIF1α silencing combined with ATRA on the growth of tumors in mice; *n* = 5 for each group. **E** The tumor volumes in each group mice of **D**. **F** Representative images of IHC staining for HIF1α in tumor of each group.

In order to verify the effect of CRABP2 on tumor drug sensitivity in vivo, we constructed corresponding mouse models. The results showed that CRABP2 silencing alone had no significant effect on tumor growth, but combined with DDP treatment, CRABP2 silencing could significantly inhibit tumor growth rate in mice (Fig. 7A), and the volume of tumor treated with knocked CRABP2 combined with DDP was the smallest (Fig. 7B). Correspondingly, the results of immunohistochemical staining showed that the tumor Ki67 proliferation index was the lowest in the knocked CRABP2 combined with DDP treatment group, while the cleaved-caspase3 expression level was the highest (Fig. 7C). In addition, we found that the deletion of CRABP2 can also down-regulate the expression of HIF1α in the xenograft tumors (Fig. 7C). Furthermore, we found that knockdown of HIF1α significantly increased the efficacy of ATRA in the treatment of tumors in vivo (Fig. 7D-F). Taken together, the above experimental results indicate that the expression level of CRABP2 can affect the sensitivity of tumor cells to chemotherapy drugs and the expression of HIF1α in vivo.

## Discussion

Ovarian cancer is a highly aggressive malignancy with poor prognosis and high mortality rate. The development of chemotherapy resistance is a major challenge in the treatment of ovarian cancer. In this study, we found that CRABP2 is highly expressed in ovarian cancer tissues and is associated with the malignancy of the tumor and the prognosis of ovarian cancer patients, and that CRABP2 expression level is related to the sensitivity of ovarian cancer cells to chemotherapy drugs. Metabolically, CRABP2 is related to the high metabolic activity in drug-resistant cells, and all-trans retinoic acid exacerbates this activity. Mechanistically, CRABP2 not only up-regulates the expression level of HIF1α, but also increases the localization of HIF1α in the nucleus. In drug-resistant ovarian cancer cells, knocking down HIF1α can block the resistance of CRABP2 to chemotherapy drugs in ovarian cancer cells. Our study shows for the first time that CRABP2 is associated with chemotherapy resistance in ovarian cancer.

Our study first investigated the role of CRABP2 in ovarian cancer prognosis and its association with chemotherapy resistance. Through the analysis of public databases and survival data, we found that CRABP2 mRNA was upregulated in drug-resistant ovarian cancer cells and was a significant risk factor for survival in ovarian cancer patients. Further analysis revealed that CRABP2 mRNA was a risk factor for OS and PFS regardless of clinical stage or pathological grade, and specifically in serous ovarian cancer patients. Our IHC results confirmed that high expression of CRABP2 in tumor tissues was associated with a poorer prognosis, and multivariate survival analysis identified CRABP2 as an independent prognostic factor for serous ovarian cancer. Additionally, CRABP2 was found to be highly expressed in ovarian cancer tissues compared to normal tissues, and its expression level correlated positively with tumor malignancy, including histopathological grade, clinical stage, and metastasis. Actually, a study has found that the exosome-related CRABP2 is upregulated and promotes cell proliferation in ovarian cancer, and CRABP2 performs better than CA125 in the diagnosis of ovarian cancer [23]. So, CRABP2 may play a crucial role in the development, progression, and chemotherapy resistance of ovarian cancer.

Further research on the molecular mechanisms involving CRABP2 could provide insights into potential therapeutic targets for ovarian cancer patients. In order to analyze the molecular mechanism of CRABP2 regulating drug resistance in ovarian cancer, we conducted enrichment analysis on gene data from TCGA database, and found that CRABP2 may be involved in the metabolic regulation of ovarian cancer cells. At present, more and more attention is being directed to the relationship of tumor metabolism heterogeneity and drug-resistance [25–27]. Drug-induced selection pressure is conducive to the emergence of specific metabolic characteristics [28, 29]. Some studies have focused on the metabolic rearrangement in ovarian cancer-resistant cells, including changes in the Warburg effect and oxidative phosphorylation levels, but the conclusions are inconsistent [30]. Some studies suggested that there is a stronger oxidative phosphorylation in ovarian cancer-resistant cells [31], and some suggested that the Warburg effect and oxidative phosphorylation are enhanced in resistant cells [32, 33]. We speculate that the reasons for the inconsistency may be different cell genetic background and different drugs. Our results showed that the overall metabolic capacities, including the activity of oxidative phosphorylation and the tricarboxylic acid cycle, were higher in drug-resistance cells of SKOV3/DDP and A2780/DDP than those of parental cells, and CRABP2 upregulates these metabolic capacities in ovarian cancer. Knocking down the expression of CRABP2 in resistant cells significantly inhibited their originally high metabolic activity. Collectively, our results suggest that CRABP2 regulates the sensitivity of tumor cells to chemotherapy by influencing the metabolic activity of ovarian cancer cells.

One important finding of our research is that CRABP2 can affect the expression and subcellular localization of HIF1α, and the effect of CRABP2 on drug sensitivity of ovarian cancer is dependent on HIF1α. Recent studies have shown that HIF1α, as a transcription factor that can be activated by hypoxic environments, plays an important role in tumor metabolism [34, 35]. HIF1α promotes the utilization of glucose and oxygen by tumor cells, and increases the activity of oxidative phosphorylation and tricarboxylic acid cycling [36]. In addition, HIF1α is also involved in the regulation of lipid metabolism and amino acid metabolism in tumor cells [37–40]. The studies also found that by interfering with the activity of HIF1α, it can inhibit the metabolic reprogramming of tumor cells and improve the sensitivity of tumors to chemotherapy drugs [41–44]. Our results demonstrate that HIF1α is highly expressed in drug-resistant cells and that knocking down CRABP2 leads to decreased HIF1α expression. Moreover, ATRA treatment increases HIF1α expression, suggesting a regulatory role of CRABP2 in HIF1α expression. Importantly, knocking down HIF1α enhances the sensitivity of cells to cisplatin, while overexpression of CRABP2 combined with HIF1α inhibitor treatment also increases cisplatin sensitivity. These findings highlight the dependence of CRABP2-mediated resistance on HIF1α expression and suggest that targeting HIF1α may overcome chemotherapy resistance in ovarian cancer. The molecular mechanism exploration revealed that CRABP2 promotes HIF1α expression by up-regulating its mRNA. Furthermore, the positive correlation between CRABP2 and HIF1α protein expression in ovarian cancer tissues, particularly in chemotherapy-insensitive tumors, further supports the clinical relevance of this interaction. Interestingly, it has been reported that CRAPB2 accelerates ubiquitination-mediated BAX degradation in gastric cancer cells, thereby alleviating mitochondrial apoptosis and promoting oxaliplatin resistance [45]. Therefore, CRAPB2 may regulate the development of drug resistance in a variety of tumors through multiple mechanisms.

In conclusion, our study provides evidence that CRABP2 is closely related to the malignancy and drug resistance of ovarian cancer. CRABP2 affects the sensitivity of ovarian cancer cells to chemotherapy drugs by increasing the overall metabolic level in these cells, and by regulating the expression of HIF1α. Our findings suggest that targeting CRABP2 and HIF1α may be a promising strategy for overcoming chemotherapy resistance in ovarian cancer. However, further studies are needed to fully elucidate the molecular mechanisms underlying the roles of CRABP2 and HIF1α in ovarian cancer.

### Supplementary information

Supplementary figure legends

Supplement Figure S1

Supplement Figure S2

Supplement Figure S3

Supplement Figure S4

Supplement Figure S5

original data files

Supplementary excel1

Academic Journals Reporting Checklist

## Data Availability

Not applicable.
