# Supplementary material for: CRABP2 affects chemotherapy resistance of ovarian cancer by regulating the expression of HIF1α
Source: Cell Death Dis. 2024 Jan 9;15(1):21. doi: 10.1038/s41419-023-06398-4 (PMC10776574; doi:10.1038/s41419-023-06398-4)
Supplement: Supplementary file 1 — Supplementary figure legends [file 41419_2023_6398_MOESM1_ESM.docx]

**Supplement Figures:**

**Fig. S1 Relationships between the expression levels of CRABP2 mRNA in ovarian cancer tissues and prognosis of patients in data of KmPlot.**

A and B, The overall survival (OS) analysis (A) and the progress free survival (PFS) analysis (B) in patients with different clinical stages. C and D, The overall survival (OS) analysis (C) and the progress free survival (PFS) analysis (D) in patients with different pathological grades. E and F, The overall survival (OS) analysis (E) and the progress free survival (PFS) analysis (F) in patients with serous ovarian cancer.

**Fig. S2 The expression of CRABP2 in ovarian cancer tissues and its relationship with clinicopathological features of patients.**

A, The expression level of CRABP2 mRNA in human individual organs and corresponding tumor tissues. B, The expression level of CRABP2 mRNA in various tumor tissues and their corresponding normal tissues. C, The expression levels of CRABP2 protein in different pathological types of ovarian cancer in the Human Protein Atlas (HPA). D, The expression levels of CRABP2 mRNA protein in different stage of ovarian cancer of GEPIA2 data. E, The expression levels of CRABP2 mRNA protein in normal ovarian tissues, ovarian cancer tissues and metastasis of GEPIA2 data.

**Fig. S3 Relationships between the expression levels of CRABP2 mRNA in ovarian cancer tissues and prognosis of patients receiving chemotherapy in data of KmPlot.**

A, The ovarian cancer patients whose chemotherapy regiments include platin. B, The ovarian cancer patients whose chemotherapy regiments include Taxol. C, The ovarian cancer patients whose chemotherapy regiments include platin and Taxol.

**Fig. S4 Effects of YC-1 on metabolic phenotypes of ovarian cancer cells.**

A-C, Effects of YC-1 on glucose uptake (A), lactate secretion (B) and ATP production (C) in ovarian cancer cells overexpressing CRABP2. D-E, Effects of YC-1 on glucose uptake (D), lactate secretion (E) and ATP production (F) in ovarian cancer drug-resistant cells treated with ATRA.

**Fig. S5 CRABP2 promotes HIF1α expression by affecting its transcription level.**

A, The effects of silencing the expression of CRABP2 on the stability of HIF1α mRNA in ovarian cancer cells treated with Actinomycin D. B, The effects of ATRA on the stability of HIF1α mRNA in ovarian cancer drug-resistant cells treated with Actinomycin D. C, The possible RARE motifs in the promoter region sequence of HIF1α. D, The relative luciferase level of HIF1α in CRABP2 knockdown and control cells. E, The relative luciferase level of HIF1α in ovarian cancer drug-resistant cells treated with ATRA.

**Supplement excel 1:** **the protein profile bound to HIF1a mRNA by using the information in the database of ENCORI (http://starbase.sysu.edu.cn).**

**Original data files**

The original images of western blot used in the article
