# Supplementary figures and images for: CRABP2 affects chemotherapy resistance of ovarian cancer by regulating the expression of HIF1α
Source: Cell Death Dis. 2024 Jan 9;15(1):21. doi: 10.1038/s41419-023-06398-4 (PMC10776574; doi:10.1038/s41419-023-06398-4)

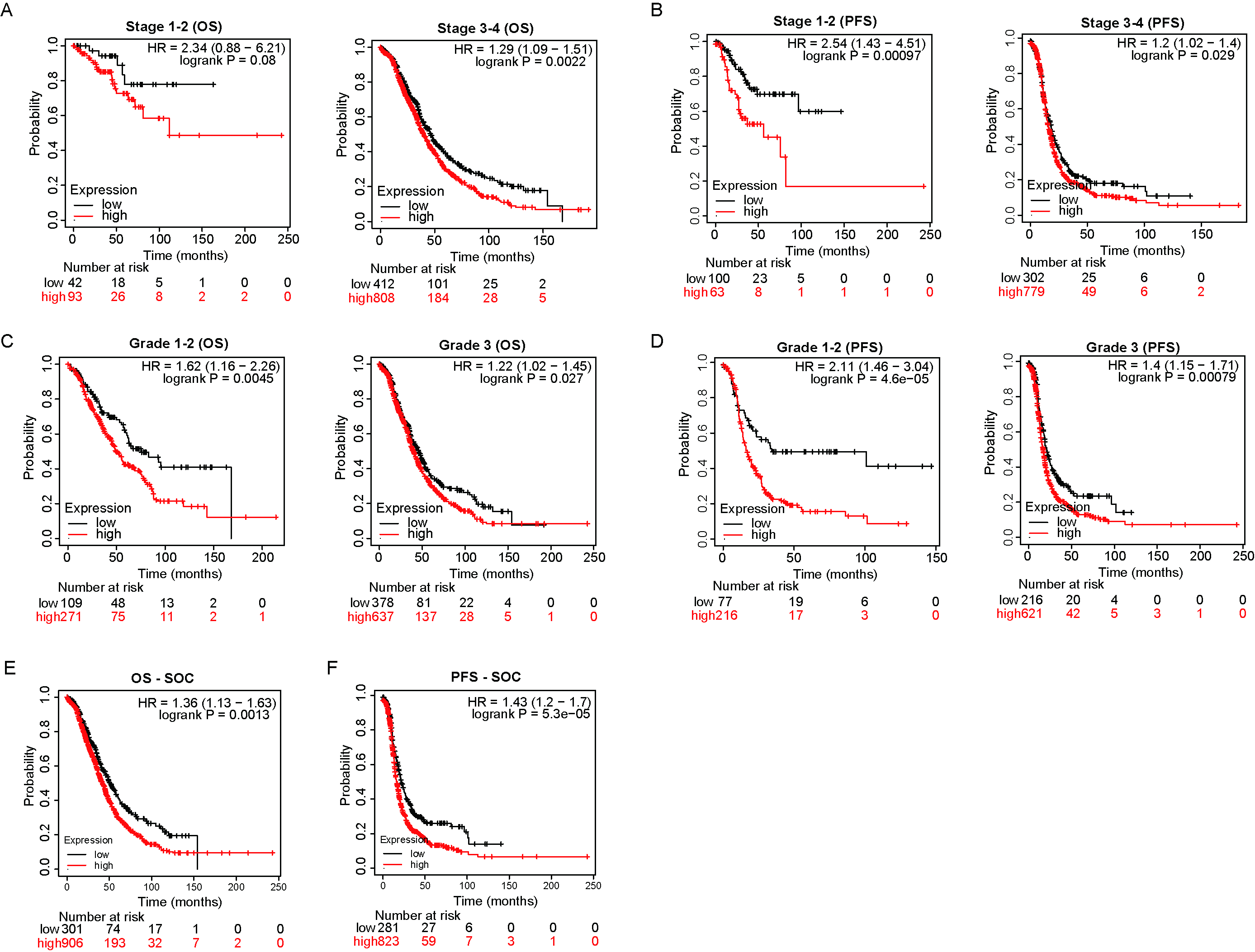

Supplement: Supplementary file 2 — Supplement Figure S1 [file 41419_2023_6398_MOESM2_ESM.tif]

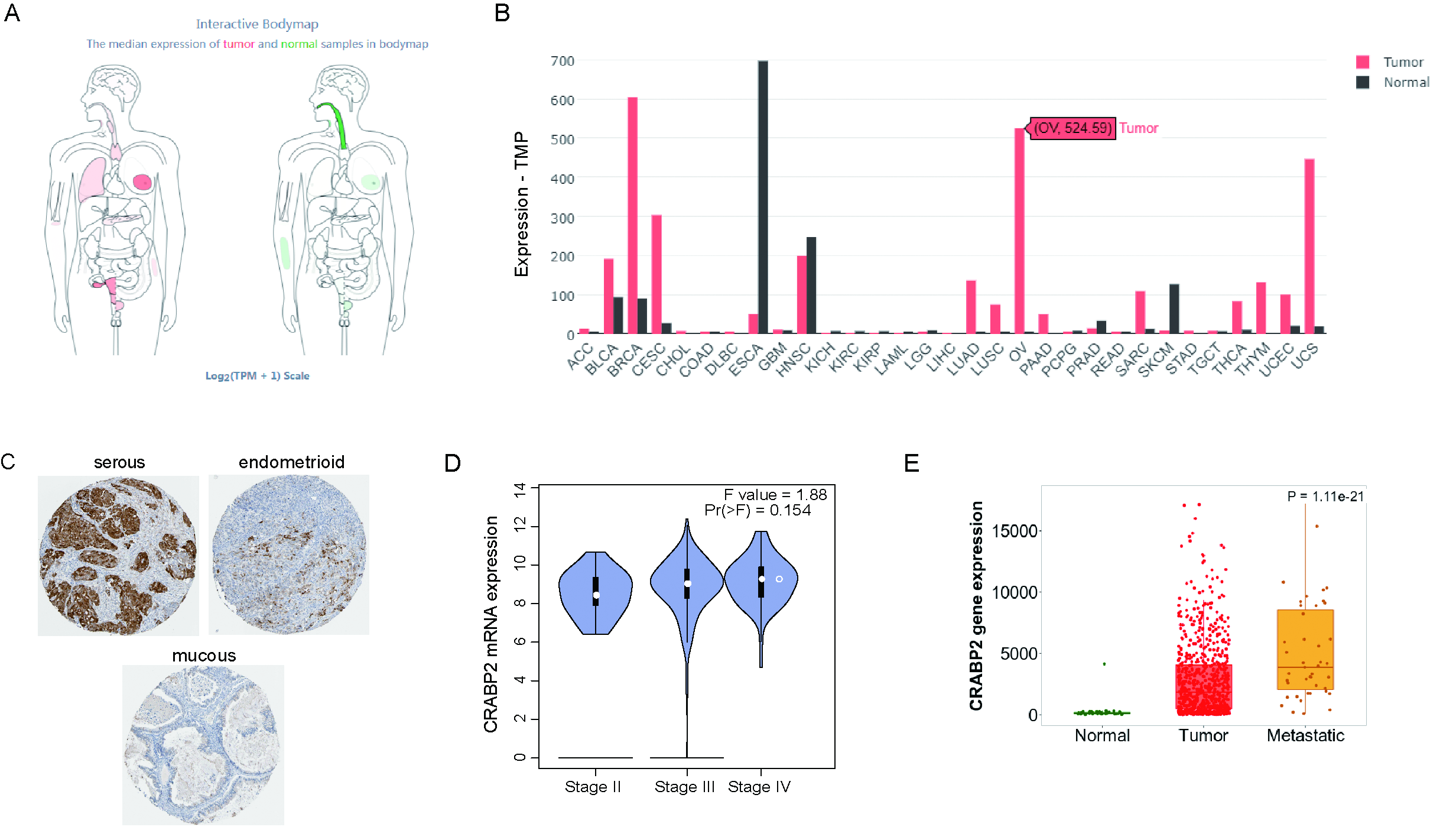

Supplement: Supplementary file 3 — Supplement Figure S2 [file 41419_2023_6398_MOESM3_ESM.tif]

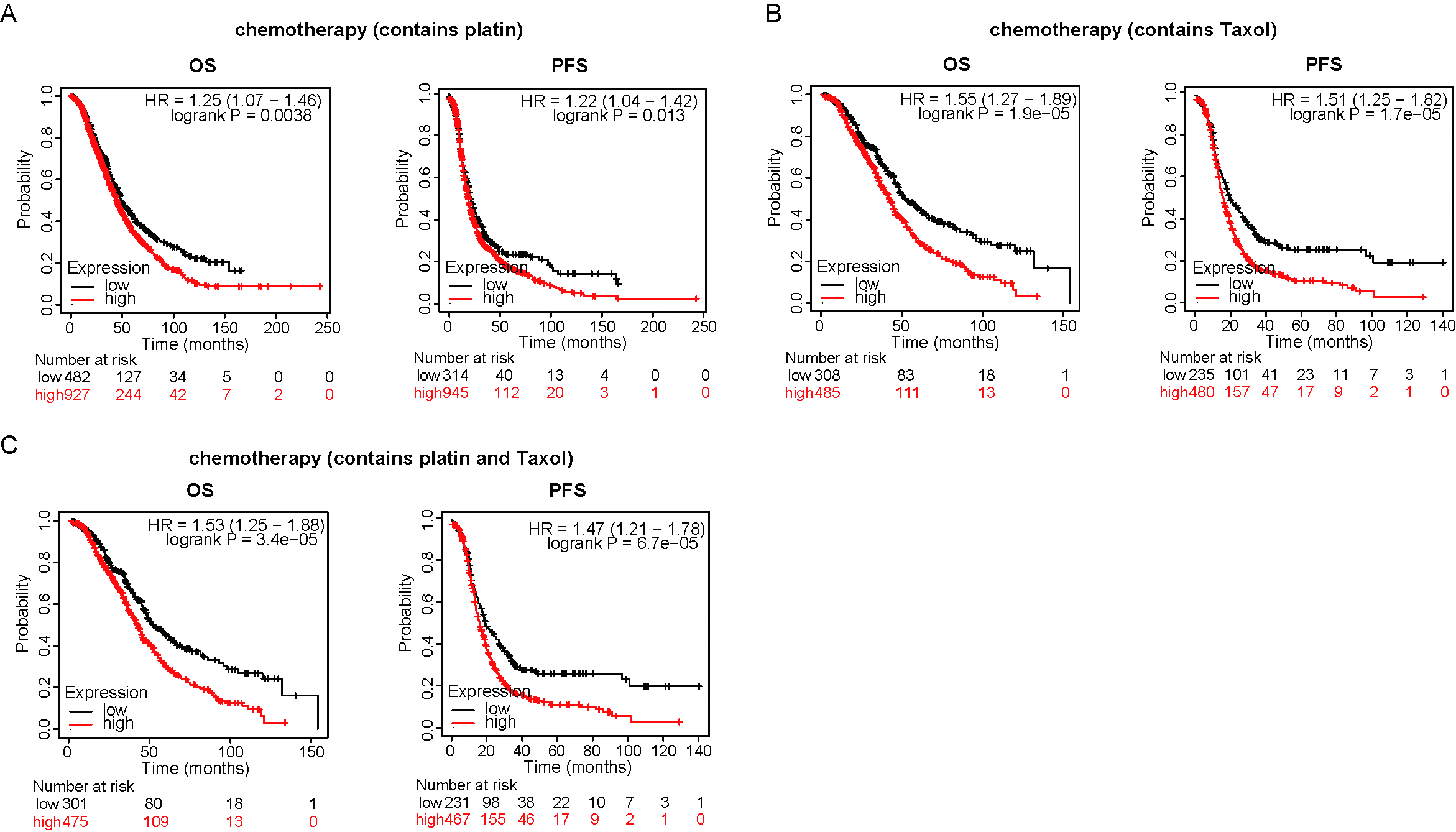

Supplement: Supplementary file 4 — Supplement Figure S3 [file 41419_2023_6398_MOESM4_ESM.tif]

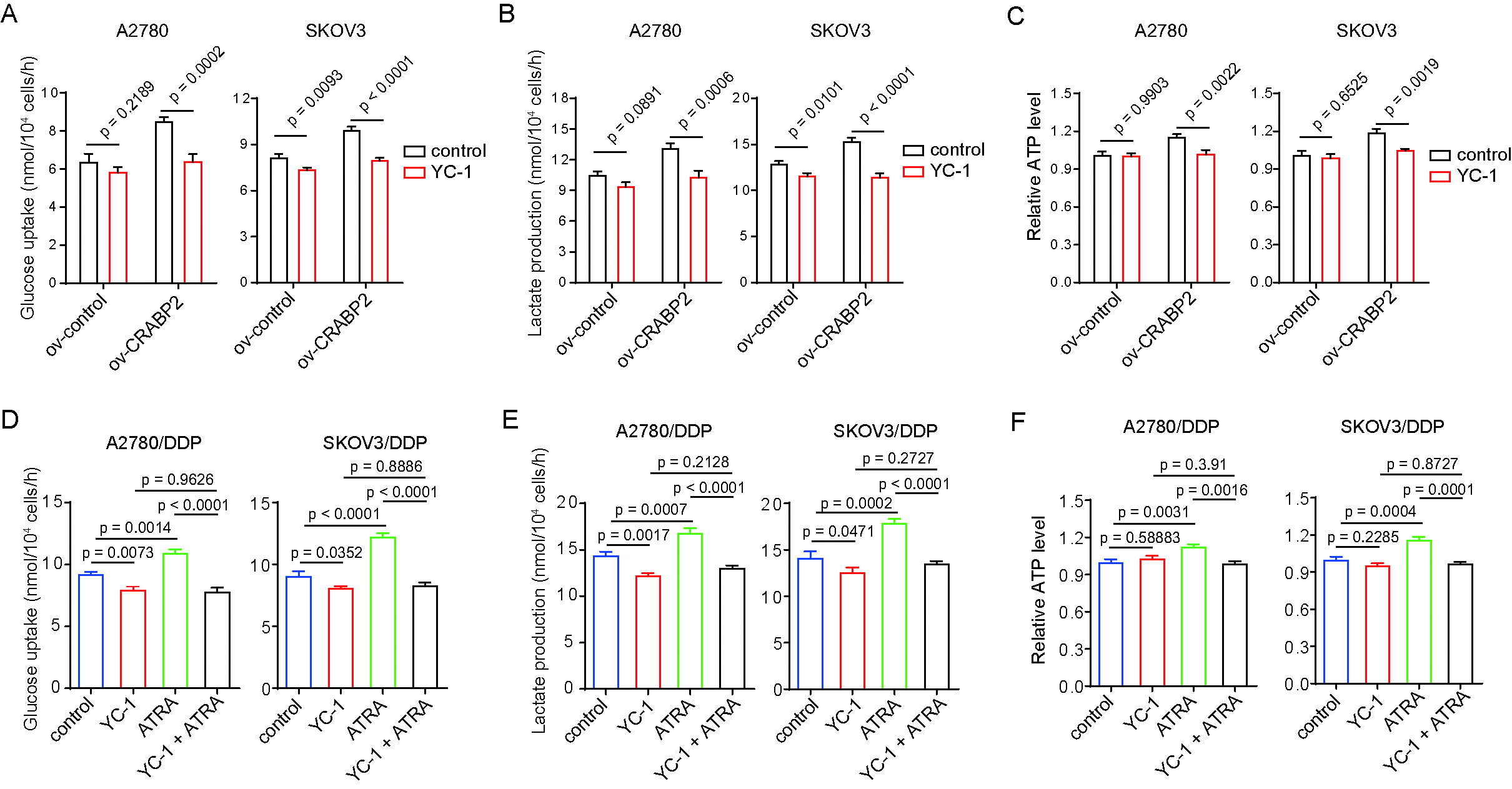

Supplement: Supplementary file 5 — Supplement Figure S4 [file 41419_2023_6398_MOESM5_ESM.tif]

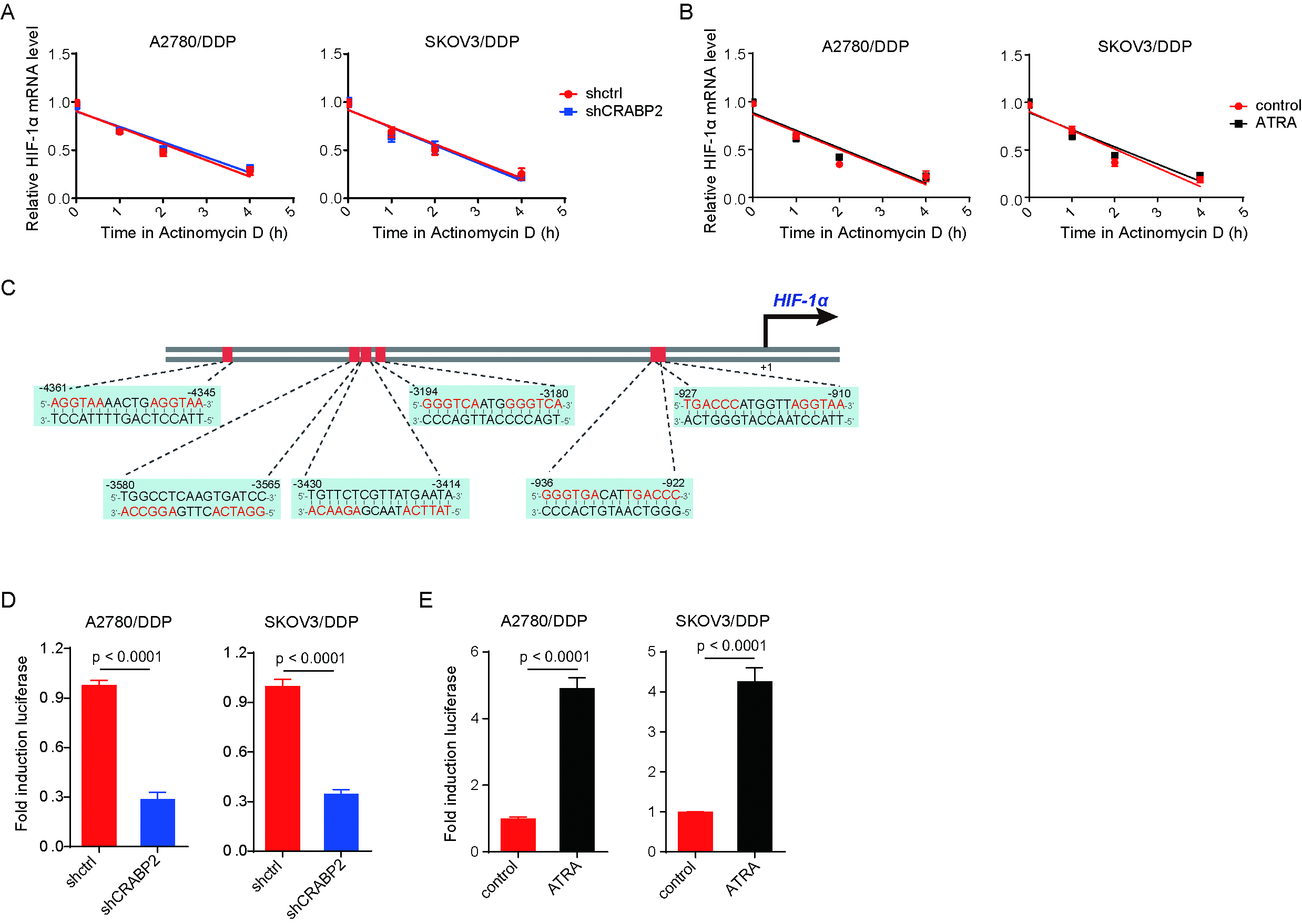

Supplement: Supplementary file 6 — Supplement Figure S5 [file 41419_2023_6398_MOESM6_ESM.tif]

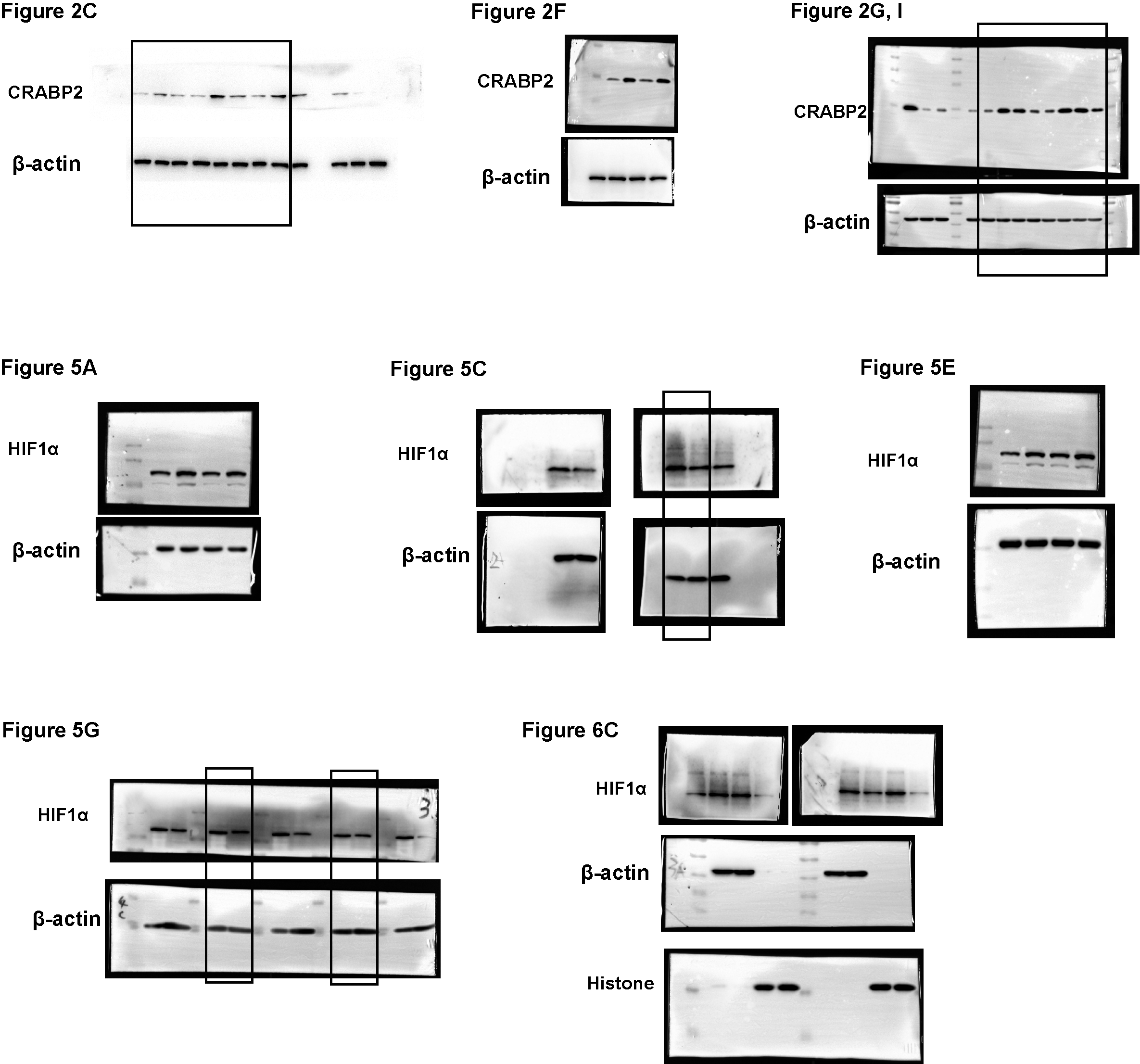

Supplement: Supplementary file 7 — original data files [file 41419_2023_6398_MOESM7_ESM.tif]
